# Mechanically reconfigurable architectured graphene for tunable plasmonic resonances

**DOI:** 10.1038/s41377-018-0002-4

**Published:** 2018-06-13

**Authors:** Pilgyu Kang, Kyoung-Ho Kim, Hong-Gyu Park, SungWoo Nam

**Affiliations:** 1 Department of Mechanical Science and Engineering, University of Illinois at Urbana-Champaign, Urbana, IL 61801 USA; 2 Department of Mechanical Engineering, George Mason University, Fairfax, VA 22030 USA; 3 Department of Physics, Korea University, Seoul, 02841 Republic of Korea; 4 KU-KIST Graduate School of Converging Science and Technology, Korea University, Seoul, 02841 Republic of Korea

## Abstract

Graphene nanostructures with complex geometries have been widely explored for plasmonic applications, as their plasmonic resonances exhibit high spatial confinement and gate tunability. However, edge effects in graphene and the narrow range over which plasmonic resonances can be tuned have limited the use of graphene in optical and optoelectronic applications. Here we present a novel approach to achieve mechanically reconfigurable and strongly resonant plasmonic structures based on crumpled graphene. Our calculations show that mechanical reconfiguration of crumpled graphene structures enables broad spectral tunability for plasmonic resonances from mid- to near-infrared, acting as a new tuning knob combined with conventional electrostatic gating. Furthermore, a continuous sheet of crumpled graphene shows strong confinement of plasmons, with a high near-field intensity enhancement of ~1 × 10^4^. Finally, decay rates for a dipole emitter are significantly enhanced in the proximity of finite-area biaxially crumpled graphene flakes. Our findings indicate that crumpled graphene provides a platform to engineer graphene-based plasmonics through broadband manipulation of strong plasmonic resonances.

## Plasmonics: Exploiting the properties of crumpled graphene

An innovative approach for making tunable plasmonic nanoparticles using crumpled graphene opens the door to new optical and optoelectronic devices. Graphene is a promising plasmonic material due to its low optical loss, high spatial confinement, and tunable plasmonic resonances. However, its structural patterns fix the resonance wavelengths, making broadband tunability across near- to mid-infrared wavelengths very challenging, limiting its range of applications. This led Pilgyu Kang from the University of Illinois at Urbana-Champaign in America, in collaboration with fellow American and South Korean colleagues, to turn to crumpled graphene, a form of graphene with unique optical and physical properties. By modelling the plasmonic resonances of crumpled graphene, the researchers demonstrated mechanically reconfigurable structures that enable a much broader range of spectral tunability, leading to new applications from optical sensors to photovoltaic devices.

## Introduction

Graphene is a promising plasmonic material with superior properties that distinguish it from conventional noble metals^1,2^, including relatively low optical loss, high spatial confinement, and tunable plasmonic resonances. Graphene-based plasmonic devices have shown potential in broad applications, including optical biosensing^3^, optical communications^4^, photodetectors^5^, and plasmonic metamaterials^6^. In particular, desired plasmonic resonances are easily excited in lithographically patterned graphene structures^4,6–14^ using a far-field coupling method. However, such structural patterns in graphene are not reconfigurable, thus fixing their resonance wavelengths. Although one can tune the resonance wavelength of graphene through electrical gating^7,11,15^, it is challenging to achieve broadband tunability across the near- to mid-infrared wavelength ranges. While graphene grating structures have been proposed for on–off switching and plasmonic modulation^16–19^, post-fabrication reconfiguration of plasmonic structures has not yet been realized.

It has recently been demonstrated that mechanical crumpling of graphene can be used to create different surface structures while maintaining reversibility^20–22^. However, the potential of crumpled graphene structures for reconfigurable plasmonic materials has not been explored. In this work, we report on the use of mechanically reconfigurable crumpled graphene structures to realize strong plasmonic resonances with broadband tunability. We introduce a resonant electrical inductor–capacitor (*LC*)-circuit model to describe the characteristics of plasmonic resonances in the crumpled graphene. By varying the geometry of the crumpled graphene structures, we show a tunable spectral range for plasmonic resonances that is fivefold broader than that achieved by conventional electrical gating. Our results show that mechanical reconfiguration of crumpled graphene serves as a new way to manipulate strong plasmonic resonances over a broad spectral range.

## Materials and methods

### Numerical simulations

**Fig. 1:**
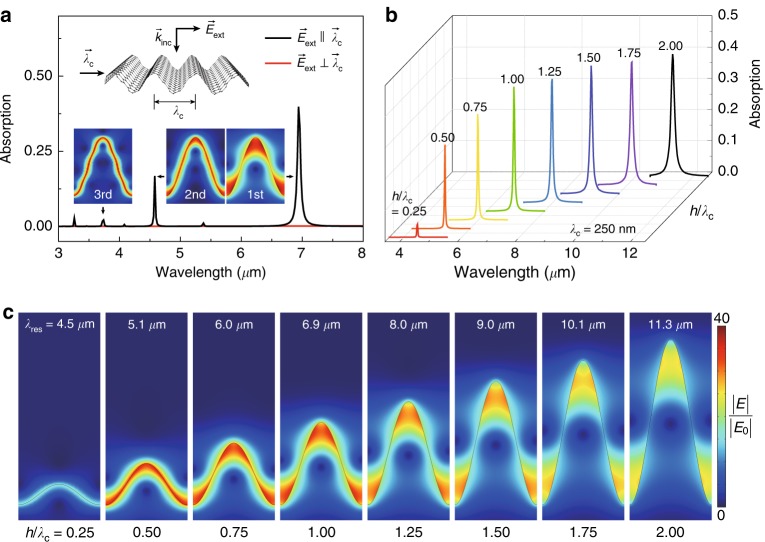
Mechanically reconfigurable plasmonic resonances of crumpled graphene structures. **a** Optical absorption spectra of a crumpled graphene structure with *λ*_c_ = 250 nm and *h*/*λ*_c_ = 1, where *λ*_c_ is the crumpled wavelength and *h* is the crumple height, for $\overrightarrow E _{\mathrm{ext}}$||$\overrightarrow \lambda _{\mathrm{c}}$ (black) and $\overrightarrow E _{\mathrm{ext}} \bot \overrightarrow \lambda _{\mathrm{c}}$ (red), where $\overrightarrow E _{\mathrm{ext}}$ is the incident electric field and $\overrightarrow \lambda _{\mathrm{c}}$ is the crumpling direction vector. Fermi energy *E*_F_ and carrier mobility *µ* are set to 0.64 eV and 10,000 cm^2^/(V·s), respectively. The upper inset shows a schematic illustration for the uniaxially crumpled graphene structure. The lower insets show field intensity distributions of the first-, second-, and third-order modes at wavelengths of 6.94, 4.58, and 3.74 μm, respectively. **b** Optical absorption spectra for the uniaxially crumpled graphene structures with varying *h*/*λ*_c_ from 0.25 to 2.0 at *λ*_c_ = 250 nm, *E*_F_ = 0.64 eV, and *µ* = 10,000 cm^2^/(V·s). **c** Near-field distributions normalized by the incident field (*E*_0_) in the uniaxially crumpled graphene structures at the plasmonic resonance wavelengths *λ*_res_ denoted in **b**

**Fig. 2:**
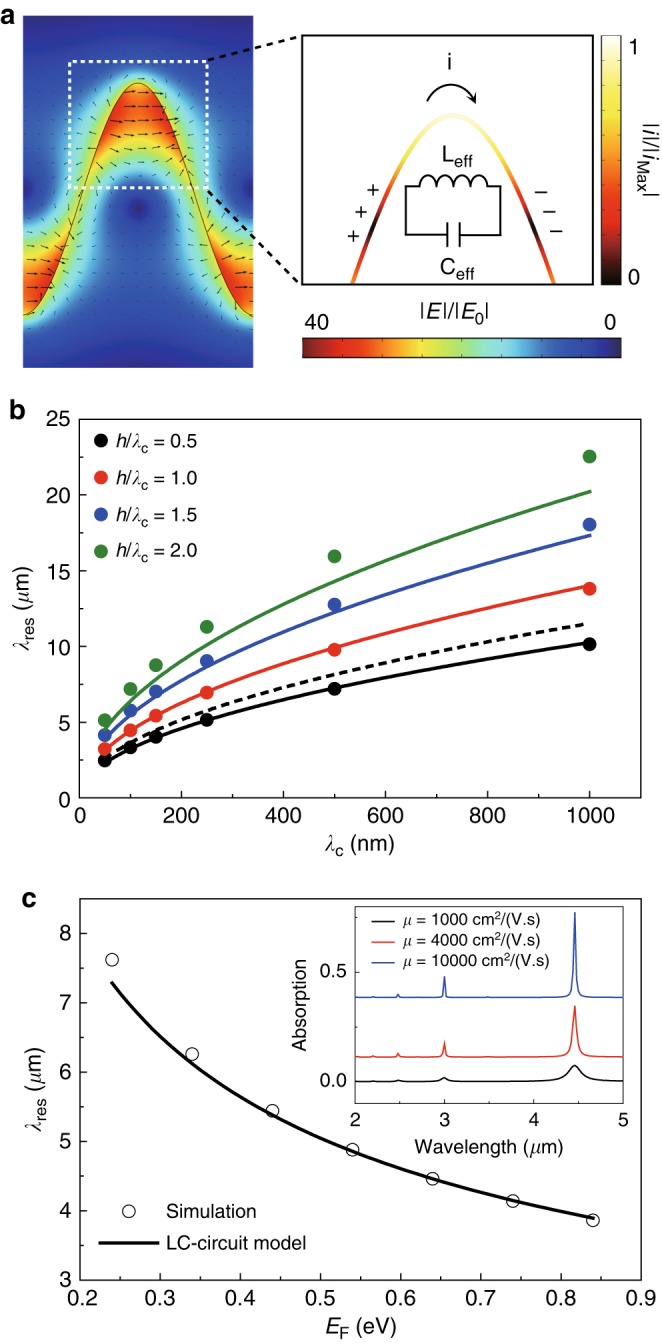
Geometry dependence of the plasmonic resonances in uniaxially crumpled graphene structures. **a** Normalized near-field distribution and electric field vectors. The right panel shows the normalized induced current distribution for the region indicated by a dotted square in the left panel. The inset of the right panel shows an *LC*-circuit model that describes the characteristics of resonant plasmons in the crumpled graphene structures. **b** Plasmonic resonance wavelengths *λ*_res_ for crumpled graphene structures (dots) with varying *λ*_c_ from 50 nm to 1 µm at constant *h*/*λ*_c_. The *h*/*λ*_c_ varies from 0.5 to 2. The curves calculated by the *LC*-circuit model (solid curves) and the conventional model (a dotted curve) are plotted together. **c** The *λ*_res_ of uniaxially crumpled graphene structures (dots) with varying *E*_F_ from 0.24 to 0.84 eV at *λ*_c_ = 100 nm, *h*/*λ*_c_ = 1, and *µ* = 10,000 cm^2^/(V·s). The *λ*_res_ was also calculated as a function of *E*_F_ using the *LC*-circuit model (solid curve). The inset shows the optical absorption spectra of uniaxially crumpled graphene structures for various *µ* from 1000 to 10,000 cm^2^/(V·s) at *E*_F_ = 0.64 eV, *λ*_c_ = 100 nm and *h*/*λ*_c_ = 1

**Fig. 3:**
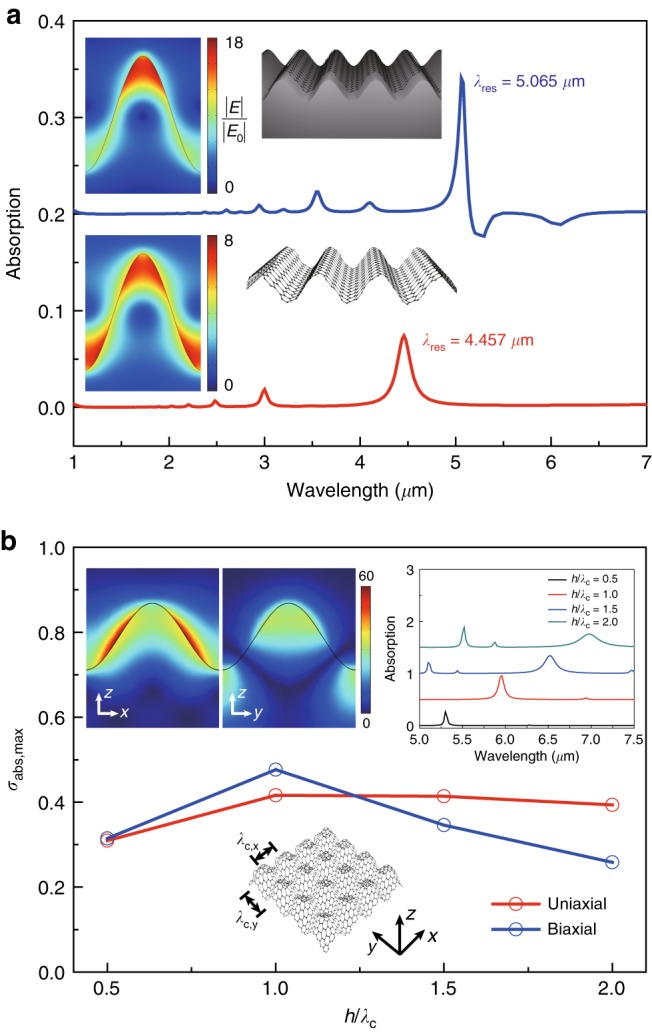
Substrate effect for uniaxially crumpled graphene structures and plasmonic resonances for biaxially crumpled graphene. **a** Optical absorption spectra of the uniaxially crumpled graphene structures with a substrate and without a substrate at *λ*_c_ = 100 nm, *h*/*λ*_c_ = 1, *E*_F_ = 0.64 eV, and *µ* = 1000 cm^2^/(V·s). The insets show schematic illustrations of the uniaxially crumpled graphene with and without a substrate together with the near-field distributions normalized by *E*_0_ at the plasmonic resonance wavelengths *λ*_res_. **b** Maximum optical absorption values *σ*_abs,max_ of uniaxially and biaxially crumpled graphene structures at the plasmonic resonance wavelengths for various *h*/*λ*_c_ from 0.5 to 2 at *λ*_c_ = 250 nm (*E*_F_ = 0.64 eV and *µ* = 10,000 cm^2^/(V·s)). The insets show optical absorption spectra (upper right) for various *h*/*λ*_c_ and a schematic illustration (bottom) of the biaxially crumpled graphene structures. The upper left insets show normalized near-field distributions (|*E*|/|*E*_0_|) for the biaxially crumpled graphene structures (*x*–*z* and *y*–*z* cross-sectional planes for *h*/*λ*_c_ = 1)

**Fig. 4:**
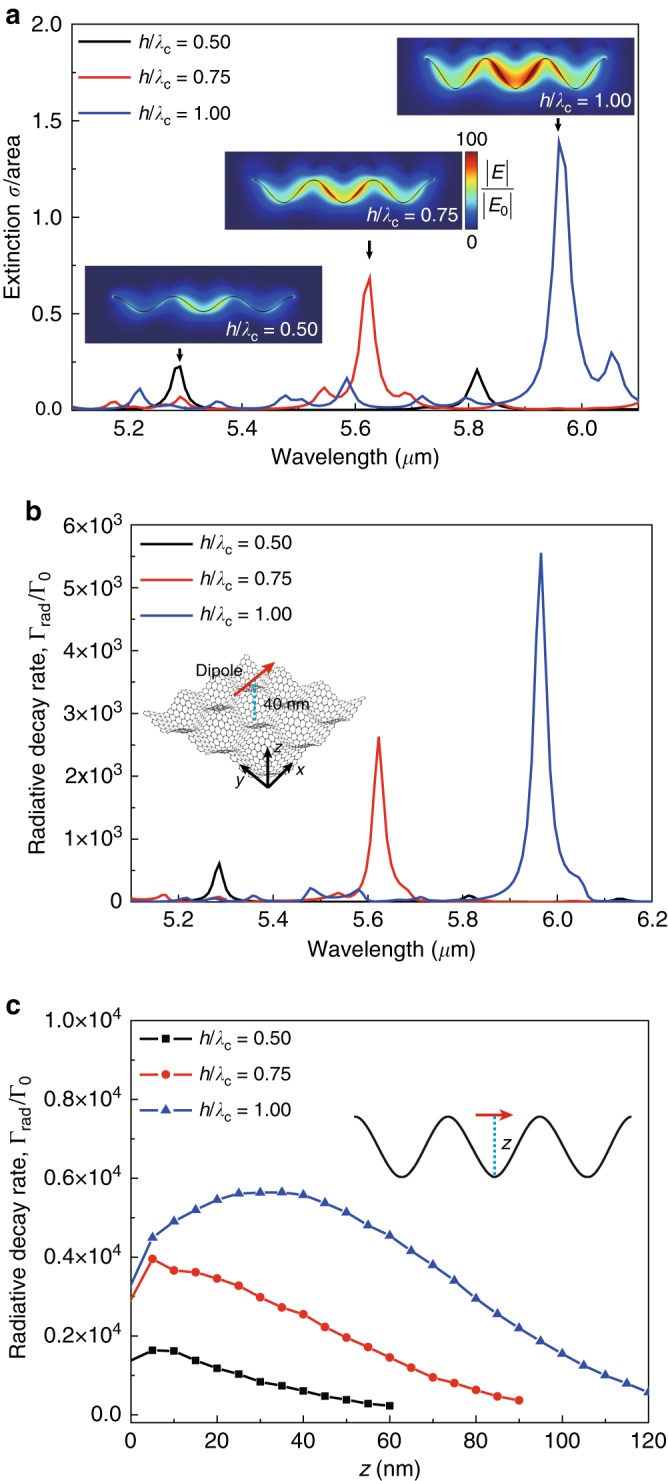
Plasmonic resonances for finite-area biaxially crumpled graphene flakes. **a** Optical extinction cross-section of the finite-area biaxially crumpled graphene structures with three periods of crumples with varying *h*/*λ*_c_ from 0.5 to 1.0 at *λ*_c_ = *λ*_c,*x*_ = *λ*_c,*y*_ = 250 nm. Normal incident light illuminated the top of the biaxially crumpled graphene flake along the *z* direction. The polarization of the incident light was along the crumples in the *x* direction. The insets show normalized near-field distributions in the biaxially crumpled graphene flakes for various *h*/*λ*_c_. **b** Radiative decay rates of the dipole emitter placed 40 nm above the center of a biaxially crumpled graphene flake for various *h*/*λ*_c_ from 0.5 to 1.0 at *λ*_c_ = 250 nm, *E*_F_ = 0.64 eV, and *µ* = 10,000 cm^2^/(V·s). The inset shows a schematic illustration for a biaxially crumpled graphene flake with a dipole emitter. The dipole moment orientation for the emitter lies parallel with the *x* crumpling direction. **c** Radiative decay rates as a function of vertical distance (*z*) between a dipole emitter and a biaxially crumpled graphene flake at the plasmonic resonances. *h*/*λ*_c_ varied from 0.5 to 1.0, with *λ*_c_ = 250 nm, *E*_F_ = 0.64 eV, and *µ* = 10,000 cm^2^/(V·s). The inset shows a schematic illustration for a biaxially crumpled graphene flake with a dipole emitter placed at various vertical distances (*z*)

We performed full-wave simulations using the finite element method (COMSOL Multiphysics, wave optics module) to investigate the plasmonic resonances of crumpled graphene structures. We modeled graphene as a two-dimensional surface current tangential to the crumpled structure: ***J***_***s***_ = *σ****E***_***t***_, where ***J***_***s***_ is the surface current, *σ* is the optical conductivity of graphene, and ***E***_***t***_ is the tangential component of the electric field to the graphene surface^23,24^. We used a mobility value of 10,000 cm^2^/(V·s), which is achievable for crumpled graphene based on the reported range of mobilities (*μ*) for flat graphene^15^ from ~1000 cm^2^/(V·s) for chemical vapor deposition grown graphene to 230,000 cm^2^/(V·s) for suspended exfoliated graphene. Moreover, the mobility of wrinkled graphene was shown to be similar to that of flat graphene^25,26^. For the optical absorption/extinction spectra of uniaxially and biaxially crumpled graphene in Figs. 1–3, we performed absorption/transmission calculations with periodic boundary conditions imposed on the respective crumpling directions. The optical extinction spectra were obtained by calculating 1−*T*/*T*_0_, where *T* and *T*_0_ are the transmittance values with and without crumpled graphene, respectively. To investigate substrate-induced effects on plasmonic resonances in Fig. 3a, a silicon oxide (SiO_2_) substrate^27^ was used as a model material. In Fig. 4a, we obtained the extinction cross-section for a normal-incident electromagnetic field by integrating over the scattered light power and optical loss in graphene. In Fig. 4b, c, we calculated the enhancement of the radiative decay rates by taking the ratio of the radiated light power from a dipole emitter with a crumpled graphene flake to that without the crumpled graphene flake. In Fig. 4, we applied the perfectly matched layer for all boundaries of the calculation domain to reduce reflections from the boundaries.

### Optical conductivity of graphene

We calculated the optical conductivity of graphene (*σ*) for varying Fermi energy (*E*_F_) and carrier mobility (*μ*) values of graphene at a constant temperature of 300 K using a random phase approximation method^23,24,28,29^. The optical conductivity is expressed by *σ*(*ω*) = *σ*_intra_(*ω*) + *σ*_inter_(*ω*), where *σ*_intra_(*ω*) and *σ*_inter_(*ω*) are the intraband and interband conductivities, respectively^23^. They are expressed as below, where *ω* is the frequency of the incident light, *e* is the charge of an electron, *τ* is the Drude relaxation rate, *T* is the temperature, *k*_B_ is the Boltzmann constant, and ℏ is the reduced Planck constant. *E*_F_ is the Fermi energy, determined by *E*_F_ = ℏ*v*_f_(*πn*)^1/2^, where *v*_f_ is the Fermi velocity of electrons (~10^6^ m/s) and *n* is the carrier density of graphene. The Drude relaxation rate *τ* is determined by *τ = µE*_F_/*ev*_f_^2^, where *µ* is the carrier mobility of graphene.

1 $$\sigma _{{\mathrm{intra}}}\left( \omega \right) = \frac{{2e^2\omega _T}}{{\pi \hbar }}\frac{i}{{\omega + i\tau ^{ - 1}}}\log \left[ {2\cosh \left( {\frac{{\omega _{\mathrm{F}}}}{{2\omega _T}}} \right)} \right]$$

2 $$\sigma _{{\mathrm{inter}}}\left( \omega \right) = \frac{{e^2}}{{4\hbar }}\left[ {H\left( {\frac{\omega }{2}} \right) + i\frac{{2\omega }}{\pi }{\int}_0^\infty {\frac{{H\left( {\frac{{\omega \prime }}{2}} \right) - H\left( {\frac{\omega }{2}} \right)}}{{\omega ^2 - \omega {\prime}^2}}d\omega \prime } } \right]$$

3 $$H(\omega ) = \frac{{\sinh (\omega /\omega _T)}}{{\cosh (\omega _{\mathrm{F}}/\omega _T) + \cosh (\omega /\omega _T)}}$$

where *ω*_F_ = *E*_F_/*ℏ* and *ω*_*T*_ = *k*_B_*T*/*ℏ*.

## Results and discussion

### Plasmonic resonances of crumpled graphene structures

To study the plasmonic resonances of the crumpled graphene structures, we modeled crumpled graphene as a continuous sinusoidal structure with geometrical parameters, including crumple wavelength (*λ*_*c*_) and crumple height (*h*) (upper inset of Fig. 1a). First, we performed full-wave optical simulations (see Materials and methods) to investigate the far-field spectral response (i.e., optical absorption spectra) of free-standing uniaxially crumpled graphene (Fig. 1a). The incident electric field ($\overrightarrow E _{\mathrm{ext}}$) is parallel and perpendicular to the crumpling direction ($\overrightarrow \lambda _{\mathrm{c}}$) ($\overrightarrow E _{\mathrm{ext}}$||$\overrightarrow \lambda _{\mathrm{c}}$ and $\overrightarrow E _{\mathrm{ext}} \bot \overrightarrow \lambda _{\mathrm{c}}$, respectively). Our simulation result including the consideration of strain effects on crumpled graphene^30–32^ was very similar to the result obtained without consideration of strain effects (Fig. S1). In the optical absorption spectrum, we observed multiple resonance peaks, including the first- and higher-order (second- and third-order) plasmonic modes (lower insets in Fig. 1a) for $\overrightarrow E _{\mathrm{ext}}$||$\overrightarrow \lambda _{\mathrm{c}}$, whereas no resonance peak was observed for $\overrightarrow E _{\mathrm{ext}} \bot \overrightarrow \lambda _{\mathrm{c}}$. These far-field resonance peaks for $\overrightarrow E _{\mathrm{ext}}$||$\overrightarrow \lambda _{\mathrm{c}}$ are attributed to resonant graphene plasmons confined in the crumpled graphene structures^9,16,33^. The most efficient coupling of the incident light to the graphene plasmon is observed in the peak of the maximum optical absorption (Fig. 1a). In subsequent investigations, we focus on this strongest plasmonic resonance.

We further investigated plasmonic resonances in crumpled graphene for various *λ*_c_ and *h*. First, we observed the optical absorption and extinction spectra for different aspect ratios of crumple height to wavelength (*h*/*λ*_c_) at a constant *λ*_c_ of 250 nm (Fig. 1b and Fig. S2). The plasmonic resonance wavelength (*λ*_res_) redshifted from ~4.5 to ~11.3 µm with increasing *h*/*λ*_c_ from 0.25 to 2.0. The optical absorption and extinction (*σ*_abs_ and *σ*_ext_) also increased with higher *h*/*λ*_c_ from 0.25 to 1.0 but showed no significant change from *h*/*λ*_c_ = 1.0 to 2.0 (Fig. S2a, c, e). Next, we varied *λ*_c_ from 50 nm to 1 μm with a fixed aspect ratio (*h*/*λ*_c_ = 1) and observed redshifts for *λ*_res_ from ~3.2 to ~13.8 µm and an increase in *σ*_abs_ and *σ*_ext_ from ~0.12 to ~0.41 and from ~0.14 to ~0.50, respectively (Fig. S2b, d, f). Moreover, near-field distributions for graphene plasmons varied with different *h*/*λ*_c_ values (Fig. 1c). An enhanced near-field intensity near the apex and valley regions was manifested with increasing *h*/*λ*_c_. We observed a maximum near-field intensity enhancement of ~40^2^ in the apex and valley regions of crumpled graphene structures at *h*/*λ*_c_ *=* 1. Taken together, these results demonstrated the tunability of both far- and near-field plasmonic resonances by the reconfiguration of crumpled graphene structures.

### Analytical description of the plasmonic resonance in crumpled graphene structures

To describe the plasmonic resonance in crumpled graphene structures, we introduced a resonant *LC*-circuit model based on the electric field and current distributions (Fig. 2a). An effective inductance *L*_eff_ and effective capacitance *C*_eff_ are used to account for the characteristics of the current and charge induced by the graphene plasmons, respectively^34,35^. By using the crumpled structure parameters, including *λ*_c_ and *h*, as well as the electronic properties of graphene, we derived *L*_eff_ and *C*_eff_ as follows:

4 $$L_{\mathrm{eff}} = \frac{{\pi \hbar ^2}}{{e^2E_{\mathrm{F}}}}\lambda _{\mathrm{c}}{\mathrm{,}}\,C_{\mathrm{eff}} = \frac{{\varepsilon _0}}{{2\alpha }}\ln \left[ {1 + \left( {1 + e^{ - \alpha }} \right)} \right]$$

where ℏ is the reduced Planck constant, *e* is the elementary charge, *ε*_0_ is the permittivity in vacuum, and *α* = *π *− 2tan^‒1^(2*h*/*λ*_c_) is the curvature factor for the crumpled structures. By using *L*_eff_ and *C*_eff_, we derived the plasmonic resonance wavelength (*λ*_res_) of the crumpled graphene expressed as *λ*_res_ = 2*πc*(*L*_eff_*C*_eff_)^1/2^ (see SI for detailed derivation).

To validate our *LC*-circuit model, we compared the value for *λ*_res_ predicted by the *LC*-circuit model with that predicted by numerical simulations. With varying *λ*_c_ from 50 nm to 1 μm and *h*/*λ*_c_ from 0.5 to 2, the *λ*_res_ obtained by the full-wave simulations (dots) agreed with that of our *LC*-circuit model (solid curves) (Fig. 2b). In contrast, conventional analytical models account for only crumple wavelengths of a periodically modulated graphene sheet^16–19^ and cannot predict changes in *λ*_res_ in response to varying *h*/*λ*_c_ (dashed curve in Fig. 2b). The *C*_eff_ changes with varying *h*/*λ*_c_ (i.e., change of *α*), leading to a shift in *λ*_res_. This implies that controlling the charge interactions on confronting graphene surfaces by varying *h*/*λ*_c_ enables the tunability of *λ*_res_ across the near- to mid-infrared wavelength ranges. The mechanically reconfigurable crumpled graphene structures are thus an excellent platform for broadband tunability of plasmonic resonances.

To compare the wavelength tunability of the plasmonic resonances by mechanically reconfigurable crumpled graphene structures to that achievable by conventional electrical gating, we studied the electrical tunability of the plasmonic resonances in uniaxially crumpled graphene by varying the electronic properties of graphene such as the Fermi energy *E*_F_ and carrier mobility *µ* (Fig. 2c). In our simulation, *λ*_res_ blueshifted from ~7.6 to ~3.9 µm with increasing *E*_F_ from 0.24 to 0.84 eV (Fig. 2c), showing the relation *λ*_res_∝ *E*_F_^‒1/2^ as predicted by our *LC*-circuit model (a solid curve in Fig. 2c). Additionally, the *σ*_abs_ increased with higher *μ* (inset of Fig. 2c). Most notably, the tunable range of the plasmonic resonance wavelengths (∆*λ*_res_) for varying *E*_F_ was ~3.7 µm, while ∆*λ*_res_ for varying *λ*_c_ was ~20.1 µm (∆*λ*_res_ = *λ*_res,max_−*λ*_res,min_ in Fig. 2b). These results show broad spectral tunability from the mid- to near-infrared by the mechanical reconfiguration of crumpled graphene structures, which allows for a new tuning knob combined with conventional electrostatic gating.

### Substrate and crumple structural effects on plasmonic resonances

Next, we investigated substrate-induced effects on the plasmonic resonances in uniaxially crumpled graphene structures. Figure 3a shows the optical absorption spectrum of free-standing crumpled graphene in comparison to that of crumpled graphene on an undulating substrate (for a comparison to crumpled graphene on a flat substrate, see Materials and methods and Fig. S3a). The *λ*_res_ of the crumpled graphene on the undulating substrate was redshifted compared to that of the free-standing crumpled graphene (5.065 µm vs. 4.457 µm). The redshift of *λ*_res_ is attributed to confinement of resonant plasmons exclusively in the apex of the crumpled graphene on the undulating substrate (the upper left inset of Fig. 3a). It is notable that the field intensity distribution profiles in the apex and valley regions are different because of the broken symmetry of the plasmonic resonance modes between the apex and valley due to the undulating subtrate^9,36^. The *σ*_ext_ and quality factor (*Q*-factor) were enhanced approximately twofold for the undulating substrate compared to the free-standing crumpled graphene (14.0 vs. 7.6% and 63.8 vs. 26.1) (Fig. S3b). However, the crumpled graphene supported on a flat substrate exhibited plasmonic resonances similar to the free-standing graphene (*λ*_res_ = 4.488 µm vs. 4.457 µm, *Q*-factors = 26.2 vs. 26.1, and *σ*_ext,max_ = 6.9 vs. 7.6%) (Fig. S3a, b). These results show that the refractive index of the substrate enables control of the charge interactions on confronting graphene surfaces. Therefore, rational choice and design of substrates enables further modulation of plasmonic resonances in mechanically reconfigurable crumpled graphene structures.

To further explore the shape-induced plasmonic resonance of crumpled graphene, we investigated plasmonic resonances in biaxially crumpled graphene (Fig. 3b). We studied a biaxially crumpled graphene structure with the same crumpled wavelengths in both crumpling directions (*λ*_c,*x*_ = *λ*_c,*y*_) (the bottom inset of Fig. 3b). The biaxial crumpling of graphene enabled a twofold stronger coupling of the incident light to plasmonic resonance modes (i.e., higher *σ*_ext_ ~1 vs. ~0.5) (Fig. S4b) and an enhancement of the near-field intensity by a factor of 1.5^2^ ( = (60/40)^2^) compared to uniaxially crumpled graphene (the upper left insets of Fig. 3b vs. the inset of Fig. S4a). Moreover, the biaxially crumpled graphene showed strong near-field enhancements on the slope (the upper left inset of Fig. 3b), whereas the uniaxially crumpled graphene exhibited strong near-field enhancements in the apex and valley regions (the inset of Fig. S4a). With varying *h*/*λ*_c_ from 0.5 to 2 for a constant *λ*_c_, the biaxially crumpled graphene yielded one third the tunable wavelength range of the plasmonic resonance (∆*λ*_res_ ~ 1.7 µm) for the uniaxially crumpled graphene (∆*λ*_res_ ~ 6.1 µm) (Fig. S4a). Notably, biaxially crumpled graphene exhibited optical absorption reaching up to ~0.46 (Fig. 3b) and *Q*-factors on the order of those in uniaxially crumpled graphene (Fig. S4c). The absorption spectra for biaxially crumpled graphene showed the same plasmonic resonance peak wavelengths at different mobilities, including at 1000, 4000 and 10,000 cm^2^/(V·s) (Fig. S5a). The *Q*-factors increased with an increase in carrier mobility (Fig. S5a; the inset of Fig. 3b vs. Fig. S5b). The biaxially crumpled graphene shows a smaller range for wavelength tunability than the uniaxial case but shows enhanced far-field coupling of the incident light for inducing plasmonic resonances, which can be useful for ultrasensitive plasmonic detection.

### Plasmonic resonances of finite-area crumpled graphene flakes

Since our studies up to this point have only dealt with infinite sheets of graphene, we explored finite-area biaxially crumpled graphene flakes patterned into hundreds of nanometer-size squares. Such crumpled graphene flakes may find use in bio-imaging and sensing. Figure 4a shows the extinction cross-section spectra for a free-standing biaxially crumpled graphene flake with three periods of crumples in both the *x* and *y* directions. Plasmonic resonance peaks were observed for various values of *h*/*λ*_c_. The near-field distributions at the plasmonic resonance peaks show that resonant plasmons were strongly confined to the center of the crumpled graphene flake (the insets of Fig. 4a). The biaxially crumpled graphene flake exhibited near-field intensity enhancements of ~1 × 10^4^, compared to ~1.5 × 10^3^ for patterned graphene nanostructures, such as nanodisk dimers^37^. We note that the extinction cross-section spectra with *μ* = 10,000 cm^2^/(V·s) showed the same plasmonic resonance peak wavelengths as those with lower mobilities, including at 1000 and 4000 cm^2^/(V·s) (Fig. S6a). Crumpled graphene flakes may provide several benefits, including enhanced coupling with an external light and strong near-field enhancements.

Finally, to investigate the light–matter interactions^38,39^ of crumpled graphene flakes, we introduce a dipole emitter placed 40 nm above the center of a free-standing biaxially crumpled graphene flake. The orientation of the dipole moment for the emitter is parallel to the *x* crumpling direction (the inset of Fig. 4b). To characterize the decay rate enhancement by crumpled graphene flakes, we obtained radiative decay rates (*Γ*_rad_) and total decay rates (*Γ*), which were normalized by those of the emitter in free space without the crumpled graphene flake (*Γ*_0_). Figure 4b shows the radiative decay rate spectra for various values of *h*/*λ*_c_. We observed significant enhancements in the radiative decay rates, up to three orders of magnitude, at the plasmonic resonance peaks (Fig. 4b) as well as maximum Purcell factors (*Γ*/*Γ*_0_) of ~1.6 × 10^5^ (Fig. S7). Our maximum Purcell factor was similar to that previously reported for graphene disks^40^. The radiative decay rates at *λ*_res_ increased by an order of magnitude from 0.6 × 10^3^ to 5.6 × 10^3^ with increasing *h*/*λ*_c_ from 0.5 to 1. These results show that the reconfiguration of crumpled graphene flakes enables tunability for the decay rate enhancements. We note that the radiative decay rate spectra with *μ* = 10,000 cm^2^/(V·s) showed the same plasmonic resonance peak wavelengths as those with a lower mobility of 1000 cm^2^/(V·s) (Fig. S6b). We also observed a change in the radiative decay rate (Fig. 4c) and total decay rate (Fig. S8) for various vertical distances from the emitter to the crumpled graphene flake between 0 and 120 nm at the plasmonic resonances. The radiative decay rates showed enhancements of three orders of magnitude over a wide range of vertical distance between 20 and 110 nm. This suggests that crumpled graphene flakes can be used to perform robust molecular sensing against the locational uncertainty of molecules. The plasmonic resonances at mid-infrared wavelengths of ~6 µm together with the large Purcell factors as well as the tunability of the plasmonic resonances show potential for ultrasensitive plasmonic detection of molecules^41^.

## Conclusions

In summary, we have demonstrated, for the first time, mechanically reconfigurable crumpled graphene exhibiting broadband tunability of strong plasmonic resonances from the mid- to near-infrared. The reconfigured crumpled graphene structures, with varying crumple wavelengths and heights, enabled the manipulation of plasmonic resonances over a tunable wavelength range five times broader than that afforded by conventional electrical tuning of graphene. We have also demonstrated efficient excitement of graphene plasmons in crumpled graphene structures by far-field coupling of light as well as strong confinement of the excited plasmons in crumpled graphene. The strong plasmonic resonances enabled optical absorption enhancements of up to ~0.46 as well as high near-field enhancements on the order of 1 × 10^4^ for biaxially crumpled graphene flakes. Furthermore, we showed strong light–matter interactions for the crumpled graphene flakes, which enabled large decay rate enhancements to yield a maximum Purcell factor of ~1.6 × 10^5^. We believe that our crumpled graphene structures, with strong and broadly tunable plasmonic resonances in the near- to mid-infrared, can find broad applications^1,42,43^, including photonic and optoelectronic devices, ultrasensitive chemical and biological sensing, photovoltaic devices, and spectroscopy.

## Electronic supplementary material

Supplementary Information

Supplementary Movie
