# Supplementary material for: Mechanically reconfigurable architectured graphene for tunable plasmonic resonances
Source: Light Sci Appl. 2018 Jun 13;7:17. doi: 10.1038/s41377-018-0002-4 (PMC6106979; doi:10.1038/s41377-018-0002-4)
Supplement: Supplementary file 1 — Supplementary Information [file 41377_2018_2_MOESM1_ESM.pdf]

Supplementary Information

Mechanically Reconfigurable Architectured Graphene for Tunable Plasmonic Resonances

Pilgyu Kang,[1,2]† Kyoung-Ho Kim,[3]† Hong-Gyu Park,[3,4]* SungWoo Nam[1]*

[1]Department of Mechanical Science and Engineering, University of Illinois at Urbana-Champaign, Urbana, IL 61801 USA.

[2]Department of Mechanical Engineering, George Mason University, Fairfax, VA 22030, USA.

[3]Department of Physics, Korea University, Seoul 02841, Republic of Korea

[4]KU-KIST Graduate School of Converging Science and Technology, Korea University, Seoul 02841, Republic of Korea

Corresponding Author E-mails: hgpark@korea.ac.kr, swnam@illinois.edu

†These authors contributed equally to this work.

Table of Contents

Figure S1. Strain effect on the plasmonic resonances in uniaxially crumpled graphene.

Figure S2. Geometry dependence of the plasmonic resonances in uniaxially crumpled graphene.

Figure S3. Substrate effects on the plasmonic resonances of uniaxially crumpled graphene.

Figure S4. Characteristics of the plasmonic resonances of uniaxially and biaxially crumpled graphene.

Figure S5. Effects of carrier mobility of graphene on the plasmonic resonances in biaxially crumpled graphene.

Figure S6. Effects of carrier mobility of graphene on the plasmonic resonances in finite-area biaxially crumpled graphene flakes.

Figure S7. Decay rates of a dipole emitter.

Figure S8. Total decay rates of a dipole as a function of vertical distance.

Figure S9. Equivalent circuit elements for resonant *LC*-circuit model.

Movie S1. Tuning of plasmonic resonances by mechanical reconfiguration of the free-standing uniaxially crumpled graphene.

Derivation of resonant *LC*-circuit model.

Supplementary Figures

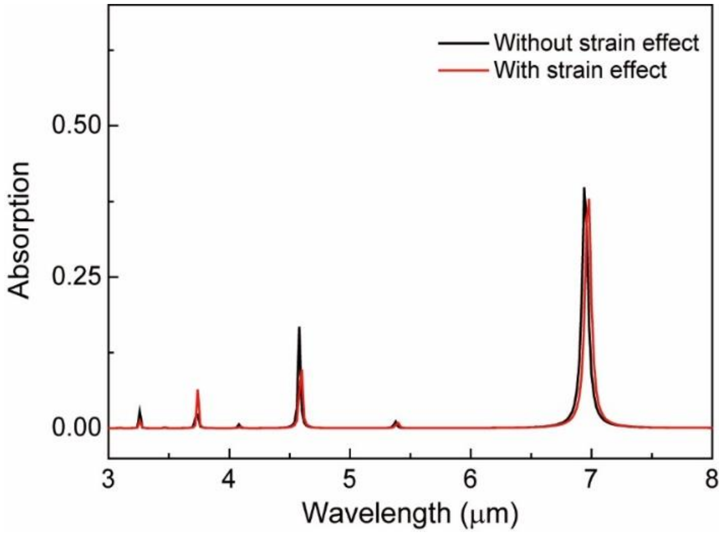

Figure S1 Strain effect on the plasmonic resonances in uniaxially crumpled graphene. The absorption spectrum of uniaxially crumpled graphene with consideration of the strain effect (red curve) was obtained by performing finite element method simulation for λ_c = 250 nm, h/λ_c = 1, μ = 10,000 $cm^2/(V \cdot s)$, and E_F = 0.64 eV. In this simulation, we used the optical conductivity considering strain effects on graphene[1–3]. To account for strains in crumpled graphene, spatially-varying strains in the crumpled graphene was obtained by calculating the radius of curvature of the crumpled graphene structure.

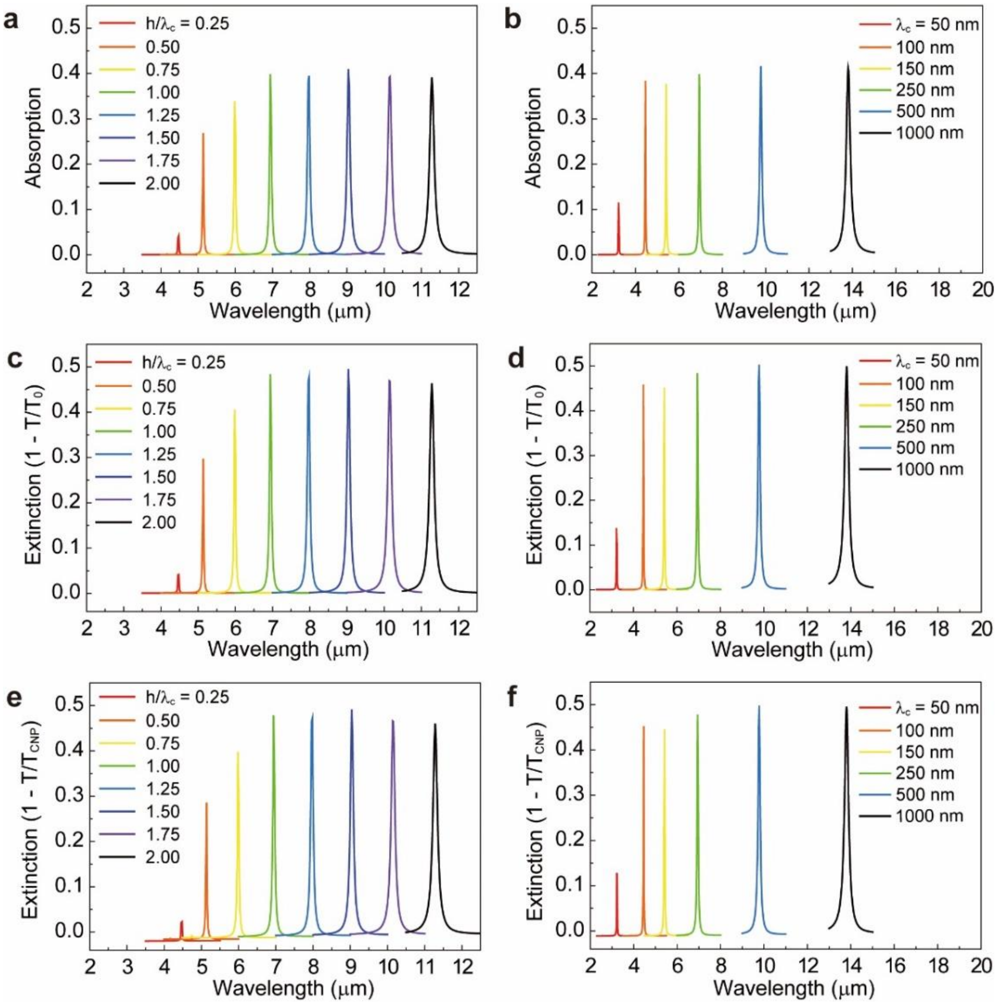

Figure S2 Geometry dependence of the plasmonic resonances in uniaxially crumpled graphene. (a, b) Optical absorption spectra, (c, d) optical extinction spectra and (e, f) optical extinction spectra normalized by the transmittance at the charge neutral point ($E_F = 0$) of uniaxially crumpled graphene with varying h/λ_c (= 0.25–2.00) at λ_c = 250 nm (a, c and e) and with varying λ_c (= 50–1,000 nm) at $h/\lambda_c = 1$ (b, d and f), where h is the crumple height and λ_c is the crumple wavelength. The Fermi energy E_F and carrier mobility μ are set to 0.64 eV and 10,000 cm^2/(V·s), respectively.

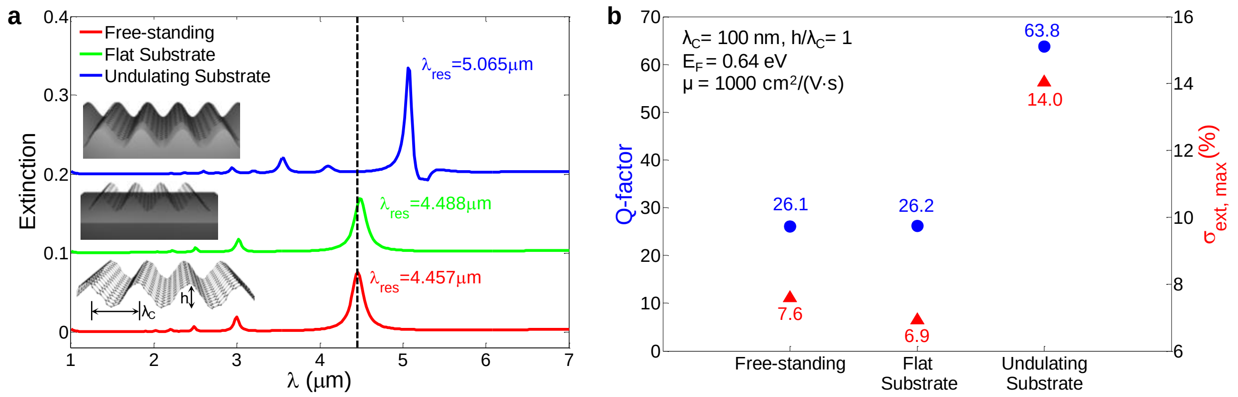

Figure S3 Substrate effects on the plasmonic resonances of uniaxially crumpled graphene. (a) Optical extinction spectra of the free-standing uniaxially crumpled graphene, uniaxially crumpled graphene on a flat substrate, and uniaxially crumpled graphene on an undulating substrate (λ_c = 100 nm, h/λ_c = 1, E_F = 0.64 eV, and μ = 1,000 cm^2/(V·s)). The insets show the schematic illustrations of crumpled graphene on an undulating substrate (top), crumpled graphene on a flat substrate (middle), and free-standing crumpled graphene (bottom). (b) Quality factor (*Q*-factor) and the maximum optical extinction values ($\sigma_{ext,max}$) of the plasmonic resonances of uniaxially crumpled graphene on the free-standing, flat and undulating substrates.

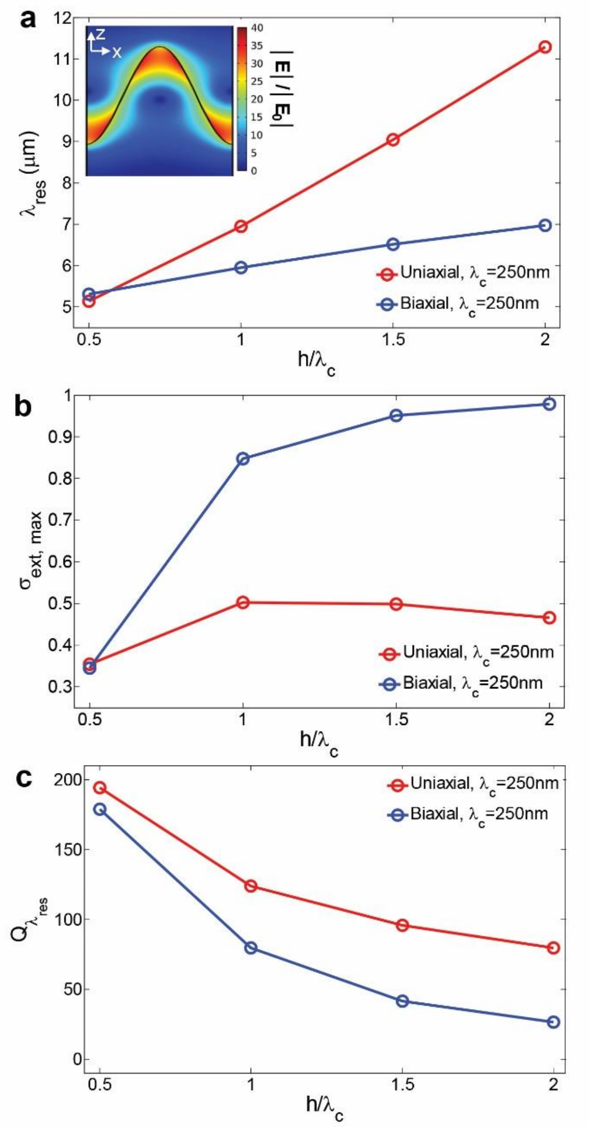

Figure S4 Characteristics of the plasmonic resonances of uniaxially and biaxially crumpled graphene. (a) Plasmonic resonance wavelength λ_{res}, (b) optical extinction $\sigma_{ext,max}$ at λ_{res}, and (c) Q-factors at λ_{res} of uniaxially and biaxially crumpled graphene. The h/λ_c varies from 0.5 to 2.0. The Fermi energy E_F and carrier mobility μ are set to 0.64 eV and 10,000 $cm^2/(V\cdot s)$, respectively.

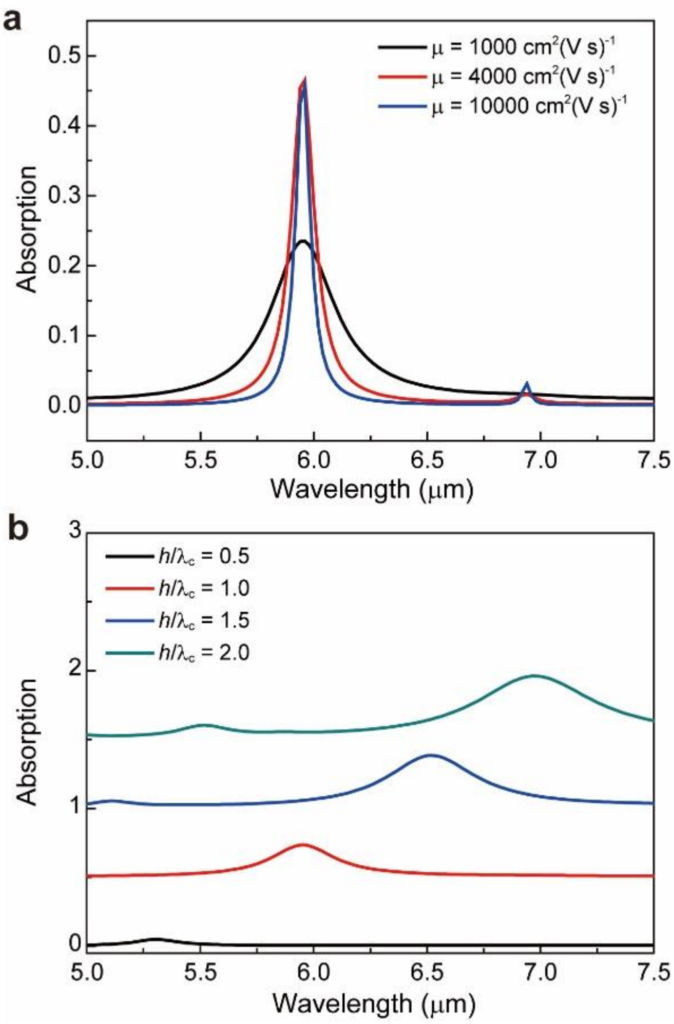

Figure S5 Effects of carrier mobility of graphene on the plasmonic resonances in biaxially crumpled graphene. (a) Optical absorption spectra of biaxially crumpled graphene for λ_c = 250 nm, h/λ_c = 1 and E_F = 0.64 eV with varying mobility of μ = 1,000, 4,000 and 10,000 $cm^2/(V{\cdot}s)$. (b) Optical absorption spectra of biaxially crumpled graphene for mobility μ = 1,000 $cm^2/(V{\cdot}s)$ (λ_c = 250 nm and E_F = 0.64 eV), with varying h/λ_c from 0.5 to 2.0.

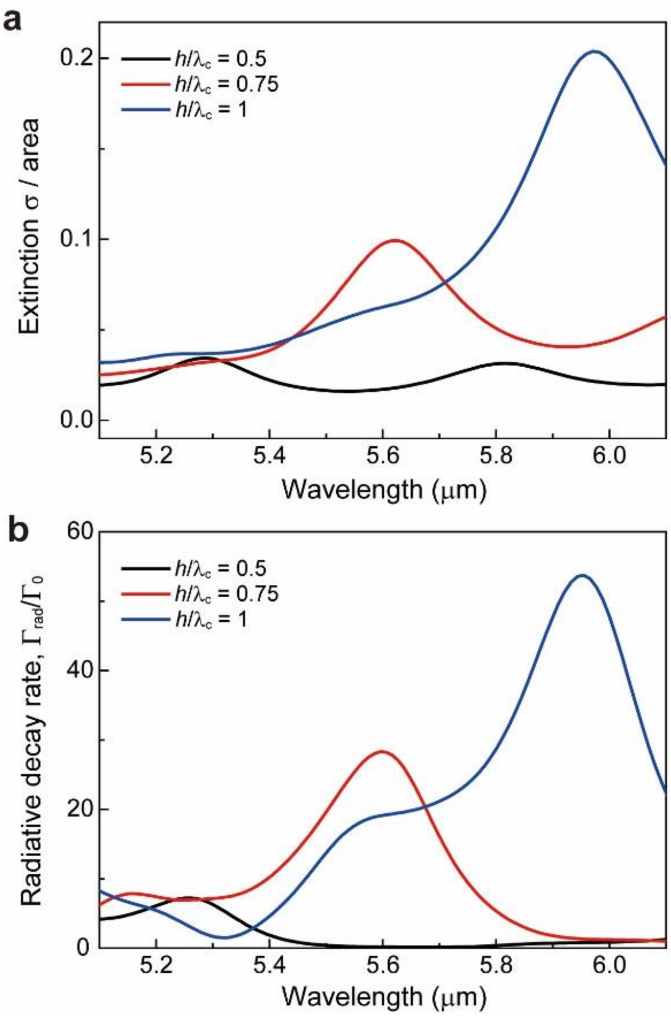

Figure S6 Effects of carrier mobility of graphene on the plasmonic resonances in finite-area biaxially crumpled graphene flakes. (a) Optical extinction cross section of the finite-area biaxially crumpled graphene structures with three periods of crumples for μ = 1,000 cm^2/(V·s). with varying h/λ_c from 0.5 to 1.0 at $\lambda_c = \lambda_{c,x} = \lambda_{c,y}$ = 250 nm. The normal incident light was illuminated on top of the biaxially crumpled graphene flake along the z-direction. The polarization direction of the incident light was parallel to the crumpling direction along the x direction. (b) Radiative decay rates of the dipole emitter placed 40 nm above the center of a biaxially crumpled graphene flake for μ = 1,000 cm^2/(V·s) with varying h/λ_c from 0.5 to 1.0 at λ_c = 250 nm and E_F = 0.64 eV.

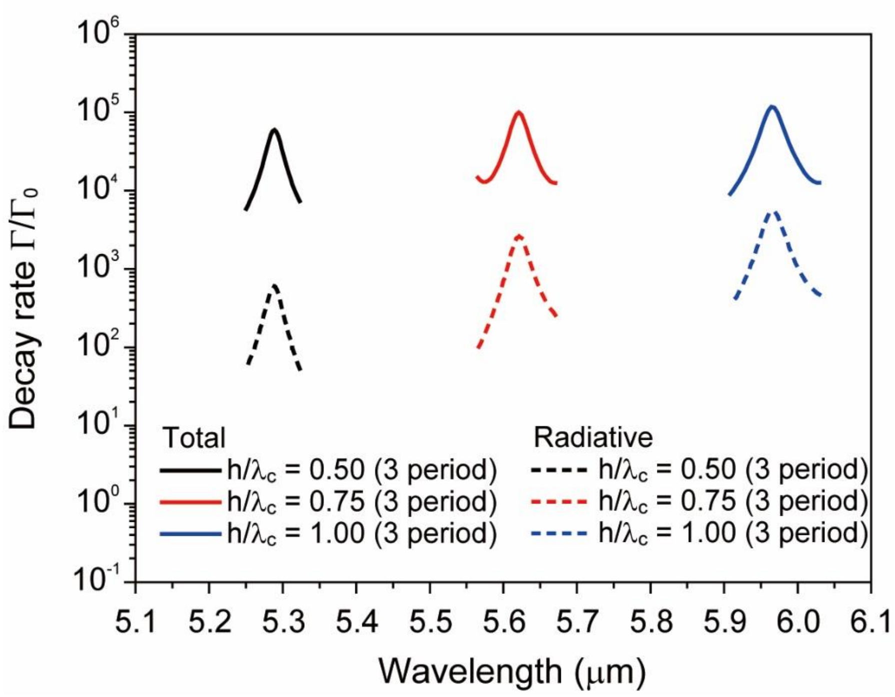

Figure S7 Decay rates of a dipole emitter. Calculated total and radiative decay rate spectra of a dipole emitter near a biaxially crumpled graphene flake, with varying h/λ_c (λ_c = 250 nm, E_F = 0.64 eV, and μ = 10,000 $cm^2/(V \cdot s)$). The dipole emitter was placed 40 nm above the center of a biaxially crumpled graphene flake. The decay rates were normalized by those of a dipole emitter in free-space without the biaxially crumpled graphene flake (Γ_0).

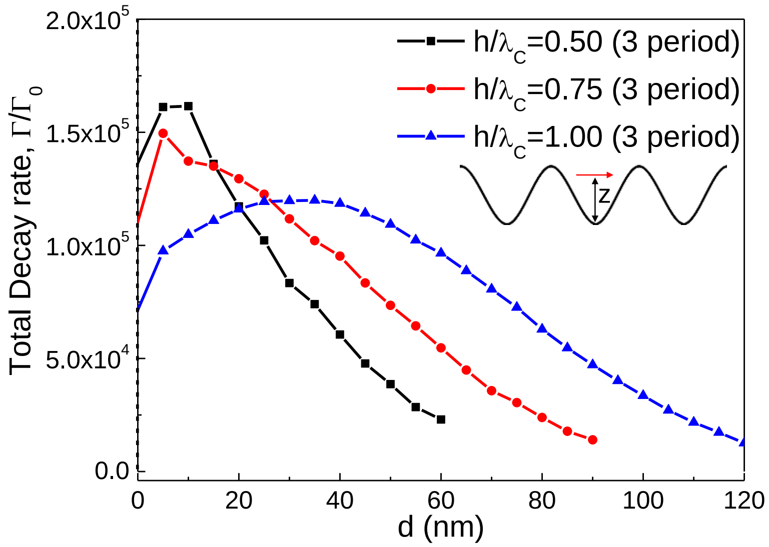

Figure S8 Total decay rates of a dipole as a function of vertical distance. Calculated total decay rates at the plasmonic resonances with varying the vertical distance (z) of a dipole emitter. The h/λ_c varies from 0.50 to 1.00 at a constant λ_c of 250 nm. The Fermi energy E_F and carrier mobility μ are set to 0.64 eV and 10,000 $cm^2/(V\cdot s)$, respectively. The decay rates were normalized by that of the emitter in free-space without the finite-area biaxially crumpled graphene (Γ_0).

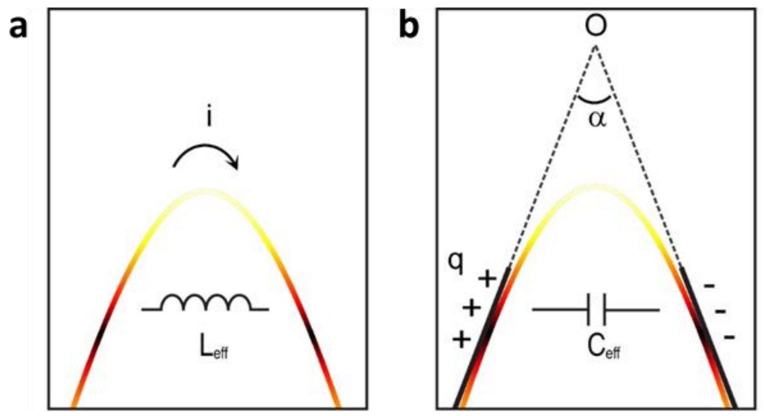

Figure S9 Equivalent circuit elements for resonant *LC*-circuit model. (a) Schematic diagram of the induced electrical current flow (i) on graphene surface by plasmonic resonance in crumpled graphene structures. Effective inductance L_{eff} is used to account for the characteristics of the induced currents by graphene plasmons. (b) Schematic diagram of the induced electric charge (q) on the slope of crumpled graphene by plasmonic resonance in crumpled graphene structures. Effective capacitance C_{eff} is used to account for the characteristics of the induced charges by graphene plasmons. The apex and valley regions are modeled as a non-parallel conducting plate capacitor (thick lines in (b)). The angle between the plates is defined by α on the origin O.

Movie S1 Tuning of plasmonic resonances by mechanical reconfiguration of free-standing uniaxially crumpled graphene. This movie shows tuning of plasmonic resonances (λ_{res} = 4.5–11.3 μm) by mechanical reconfiguration of the free-standing uniaxially crumpled graphene. The h/λ_c varies from 0.25 to 2 at λ_c = 250 nm. The insets show optical extinction spectra (upper right panel) and near-field distributions (lower right panel) of the uniaxially crumpled graphene when the TE polarized light is incident. The near-field distributions were normalized by the incident field.

Derivation of resonant *LC*-circuit model

The excited graphene plasmon induces the electrical current flow on graphene surface and the electric charge accumulation on the slope of crumpled graphene (Fig. S9). Based on the induced current and charge, the plasmonic resonance in crumpled graphene can be described by a simple resonant *LC*-circuit model with equivalent circuit elements, including inductors with inductance L_{eff} and capacitors with capacitance C_{eff}. For uniaxial crumpled graphene structures, we derived the expression

$$L_{eff} = -\frac{\lambda_c}{\omega}\mathrm{Im}\left[\frac{1}{\sigma(\omega)}\right] \quad (1)$$

for the inductance per unit length, where λ_c is the crumple wavelength and $\sigma(\omega)$ is the optical conductivity of graphene (Fig. S9a)[4]. For highly doped graphene, we derived the expression

$$\sigma(\omega) \approx \frac{e^2 E_F}{\pi\hbar^2}\frac{i}{\omega} \quad (2)$$

for the conductivity of graphene which is mainly determined by Drude response for the intraband transition in mid-infrared regime, where e is the elementary charge of an electron, $\hbar$ is the reduced Planck constant, and E_F is the Fermi energy[5]. We finally derived the expression

$$L_{eff} = \frac{\pi\hbar^2}{e^2 E_F}\lambda_c . \quad (3)$$

for the inductance as a function of crumple wavelength (λ_c). Next, we derived the expression for the capacitance per unit length in one period of the crumpled graphene structure by modeling the apex or valley regions as a non-parallel conducting plate capacitor (Fig. S9b). The non-parallel plate capacitor has an angle between plates, $\alpha = \pi - 2\tan^{-1}(2h/\lambda_c)$ which defines the curvature of the crumpled structure (Fig. S9b). Using α, we derived the expression

$$C_{plate} = \frac{\varepsilon}{\alpha} \ln\left(1 + \frac{l}{d}\right) \quad (4)$$

for the capacitance of the non-parallel plate capacitor, where ε is the permittivity of a filled material between two plates, l is the length of the plate and d is the distance from origin (O) to the edge of the plates. Then, we empirically determined the geometrical relationship of $l/d = 1 + e^{-\alpha}$ based on the electric field intensity profile near the apex or valley. In order to account for the interaction of plasmonic resonance modes between the apex and valley regions, we modeled that the capacitors in the apex and valley regions are serially connected. Then, we obtained the total capacitance of a free standing crumpled graphene structure expressed in

$$C_{eff} = \frac{\varepsilon_0}{2\alpha} \ln\left[1 + \left(1 + e^{-\alpha}\right)\right]. \quad (5)$$

The characteristic impedance of crumpled graphene structure is expressed in $1/Z_{tot} = 1/(i\omega L_{eff}) + i\omega C_{eff}$. Based on the fact that the resonance occurs when the characteristic impedance goes to infinity, we derived the resonance wavelength expressed as $\lambda_{res} = 2\pi c(L_{eff}\,C_{eff})^{1/2}$, where c is the speed of light in vacuum.

Supplementary References

1. Oliva-Leyva M, Naumis GG. Understanding electron behavior in strained graphene as a reciprocal space distortion. *Phys Rev B* 2013; **88:** 085430.

2. Oliva-Leyva M, Naumis GG. Anisotropic AC conductivity of strained graphene. *J Phys Condens Matt* 2014; **26:** 125302.

3. Ma ZH, Cai W, Xiang YX, Ren MX, Zhang XZ *et al*. Dynamic spontaneous emission control of an optical emitter coupled to plasmons in strained graphene. *Opt Express* 2017; **25:** 23070-23081.

4. Zhao B, Zhang ZM. Study of magnetic polaritons in deep gratings for thermal emission control. *J Quant Spectrosc Radiat Transfer* 2014; **135:** 81-89.

5. Koppens FHL, Chang DE, García de Abajo FJ. Graphene plasmonics: a platform for strong light-matter interactions. *Nano Lett* 2011; **11:** 3370-3377.
